# DHFR2 RNA directly regulates dihydrofolate reductase and its expression level impacts folate one carbon metabolism

**DOI:** 10.1096/fj.202401039RR

**Published:** 2025-02-17

**Authors:** Paola Drago, Niamh Bookey, Kit‐Yi Leung, Michael Henry, Paula Meleady, Nicholas D. E. Greene, Anne Parle‐McDermott

**Affiliations:** ^1^ School of Biotechnology Dublin City University Dublin 9 Ireland; ^2^ DCU Life Sciences Institute Dublin City University Dublin 9 Ireland; ^3^ Developmental Biology and Cancer Department UCL Great Ormond Street Institute of Child Health, University College London London UK

**Keywords:** DHFR, DHFR2, lncRNA, regulation, thymidylate

## Abstract

Dihydrofolate reductase activity is required in One Carbon Metabolism to ensure that the biologically active form of folate, tetrahydrofolate, is replenished and available as an enzyme cofactor for numerous cellular reactions, including purine and pyrimidine synthesis. Most cellular enzyme activity was thought to arise from the product of the *DHFR* gene on chromosome 5, with its paralogue *DHFR2* (formerly known as DHFRL1; [chromosome 3]), believed to be responsible for mitochondrial dihydrofolate activity based on recombinant versions of the enzyme. In this paper, we confirm our earlier findings that dihydrofolate reductase activity in mitochondria is derived from the *DHFR* gene rather than *DHFR2* and that endogenous DHFR2 protein is not detectable in most cells and tissues. Using HepG2 cell lines with modulated expression of either DHFR or DHFR2, we observed an impact of *DHFR2* RNA on One Carbon Metabolism mediated through an influence on DHFR expression and activity. Knockout of *DHFR2* results in a drop in dihydrofolate reductase activity, lowered 10‐formyltetrahydrofolate abundance, downregulation of *DHFR* mRNA, and diminished DHFR protein abundance. We also observed downregulation of Serine Hydroxymethyltransferase and Thymidylate Synthase, two One Carbon Metabolism enzymes that work with DHFR to support de novo thymidylate synthesis. The expression of recombinant DHFR2 resulted in restoration of DHFR mRNA and protein levels while a DHFR knockdown cell line showed upregulation of DHFR2 RNA. We propose that the *DHFR2* gene encodes an RNA molecule that regulates cellular dihydrofolate reductase activity through its impact on DHFR mRNA and protein.

## Graphical abstract

The main product of the human DHFR2 gene is RNA that functions as a regulator of its parental paralogue DHFR, at the mRNA and protein level. Dihydrofolate reductase (DHFR) is essential for One Carbon Metabolism, replenishing tetrahydrofolate as a cofactor for various cellular reactions, including nucleotide synthesis. Knockout of DHFR2 decreased DHFR expression, and related metabolites, while recombinant DHFR2 restored DHFR mRNA and protein levels. We propose DHFR2 encodes an RNA that regulates cellular DHFR activity, influencing One Carbon Metabolism.

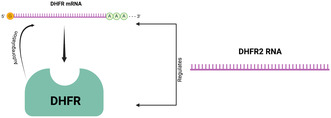

## 1 INTRODUCTION

*Dihydrofolate reductase 2* (*DHFR2*) is a retrogene of the human dihydrofolate reductase gene family that was originally proposed to be the reductase enzyme in the mitochondria to facilitate on‐site de novo thymidylate synthesis.^1^, ^2^ It was previously annotated as *DHFRL1* (dihydrofolate reductase like‐1) or *DHFRP4* (dihydrofolate reductase pseudogene 4); however, DHFR2 is now the approved symbol of the Human Gene Nomenclature Committee (genenames.org). Its identification was significant given that its parental paralogue, DHFR, plays a central role in folate One Carbon Metabolism (OCM),^3^ including being the only enzyme that can reduce dihydrofolate to tetrahydrofolate.^4^ This reductase activity ensures that one carbon unit in this pathway is recycled and available for further reactions; DHFR is also solely responsible for reducing the synthetic form of folate, that is, folic acid, in a two‐step reaction, to facilitate its bioavailability.^5^, ^6^, ^7^ This is particularly relevant during the periconceptional period of pregnancy due to its known role in the prevention of neural tube defects (NTDs).^8^ DHFR is also an established drug target, with methotrexate being one of the most widely used DHFR inhibitors that is still in clinical use for the treatment of rheumatoid arthritis and acute lymphoblastic leukemia.^9^, ^10^, ^11^, ^12^, ^13^

We, and others, have reported the presence of dihydrofolate reductase enzyme activity in purified mitochondria of human^1^, ^14^ and rodent tissue or their derived cell lines.^15^, ^16^ However, we demonstrated that in rodents, this activity is derived from DHFR and not a duplicate expressed pseudogene.^16^ This finding prompted us to reconsider the identity of *endogenous* reductase activity in the mitochondria of human cells, as our original experiments, which had pinpointed DHFR2 as the mitochondrial reductase, were based on recombinant versions of the enzyme.^2^ Moreover, the 92% amino acid sequence identity between DHFR and DHFR2^2^ makes distinguishing between the two proteins challenging, particularly when using antibodies that are likely to cross‐react with both proteins.^17^, ^18^ A targeted proteomics approach identified that the mitochondrial reductase in human cells is likely to be DHFR and not DHFR2.^14^ This called into question the functional role of the *DHFR2* gene and indicated that although the Open Reading Frame indicated a dihydrofolate reductase‐like sequence as the obvious function for this gene, the apparent lack of a detectable endogenous DHFR2 protein suggested that its function may lay beyond that of a second reductase enzyme. We also found that the recombinant DHFR2 enzyme had an approximately tenfold lower Km for the substrate dihydrofolate compared to DHFR.^2^ This enzyme kinetic difference is likely to be due to one or all of the three key amino acid differences with DHFR.^2^ These data led us to reconsider the functional role of the *DHFR2* gene and to investigate what role, if any, it might play in folate OCM.

In this paper, we sought to confirm whether DHFR, and not DHFR2, is the origin of reductase activity in the mitochondria of human‐derived cells and to elucidate the functional role of the human *DHFR2* gene using a series of cell line models with a modulated expression of DHFR and DHFR2. We present evidence that the *DHFR2* gene function lies at the RNA level producing a long noncoding RNA (lncRNA) that directly regulates DHFR and ultimately impacts OCM and de novo Thymidylate synthesis.

## 2 MATERIALS AND METHODS

### 2.1 Cell culture

HepG2 cells were purchased from the American Type Culture Collection. Cells were cultured in DMEM hi‐glucose (Gibco), supplemented with 10% FBS (Gibco) at 37°C, in a 5% CO_2_ incubator. When specified, they were supplemented with nonessential amino acids 1% (Gibco) (composition: Glycine, l‐Alanine, l‐Asparagine, l‐Aspartic acid, l‐Glutamic Acid, l‐Proline, l‐Serine—10 mM each) and HT Supplement 1% (Gibco) (sodium hypoxanthine 10 mM and thymidine 1.6 mM); abbreviated as HT/NEAA in Supporting Information. HepG2 cells are a commercially available hepatocyte carcinoma cell line and experimentation with such lines does not require institutional ethics approval. Institutional biological safety approval was received for all experiments performed.

### 2.2 Generation of the DHFR2 knockout line

The HepG2 DHFR2 knockout was obtained through the excision of the entire ORF via a double‐cut strategy employing HiFi Cas9 Ribonucleoprotein (IDT). Two separate RNPs (HiFi Cas9 + tracrRNA + crRNA [5’ DHFR2: TCAATATACGTACATGCTAT], [3’ DHFR2: CCTGGAACTTGCTATTGAGT]) targeting the extremities of the DHFR2 ORF were assembled according to the IDT protocol and reverse transfected with Lipofectamine RNAiMax (Invitrogen) and OptiMEM (Gibco). Monoclonal lines were isolated using single‐cell cloning and screened for positive transformants via PCR (Primers: DHFR2 Fb, DHFR2 Re) and RT‐PCR (Primers: FPDHFRL1Var1&2, RPDHFRL1Var1&2). We can rule out CRISPR‐Cas9 off‐target knockout of the parental DHFR gene as the knockout design was in the flanking regions of the DHFR2 ORF with no sequence homology with the genomic region of the DHFR gene.

### 2.3 Generation of the DHFR knock‐down line

The HepG2 DHFR knock‐down line was generated in line with the PITCh strategy^19^ using CRISPR‐Cas9 to facilitate the insertion of the EGFP‐2A‐Puro cassette at the end of DHFR exon 1, causing gene disruption. Two constructs, All‐in‐one and μΩ vectors, were cotransfected with the help of TransIT‐X2 (Mirus). A 4:1 TransIT‐X2:DNA ratio was used, with DNA split as an All‐in‐One:μΩ vector 2:1 ratio. Puromycin selection was performed 72 h post‐transfection at low, optimal, and high concentrations (1, 1.5, and 2 μg/mL). Cassette insertion was confirmed by PCR amplification of the two extremities (Primers: DHFR KO Fwd, Invert cassette Rev., Invert cassette Fwd, DHFR KO Rev) using Q5 High‐Fidelity DNA Polymerase (New England BioLabs). Single‐cell cloning allowed the isolation of monoclonal lines, which were further screened via PCR and RT‐PCR (Primers: DHFR Mains Fwd, DHFR Mains Rev). Finally, the presence and activity of the DHFR enzyme were assessed via Western Blot and DHFR Enzymatic Activity Assay (Abcam).

### 2.4 Constructs

The All‐in‐One vector, containing the Cas9 gene and two gRNAs (one targeting DHFR and the other targeting the gene cassette), was built in two sequential molecular cloning procedures. In the first step, the DHFR‐specific gRNA (5′‐CGGCCCGGCAGATACCTGAG‐3′) was cloned into the pX330A‐1x2 plasmid (#58766, Addgene) using BbsI restriction sites. A second cycle of digestion/ligation (Golden Gate assembly) was applied to the above vector and pX330S‐2‐PITCh (#63670, Addgene) using BsaI‐HF and QuickLigase (New England BioLabs). The positive transformants were isolated via blue/white selection. The μΩ vector was built using pCRIS‐PITChv2‐FBL (#63672, Addgene) as a template, maintaining the EGFP‐2A‐Puro gene cassette and substituting the flanking regions with DHFR microhomologies. Two sets of primers were used to amplify the vector backbone (5#‐rev DonorVector and 3#‐fwd DonorVector) and amplify the gene cassette with DHFR‐specific extremities (5Fwd DonorVect Mo and 3rev DonorVect Mo). The two DNA portions were ligated via In‐Fusion Cloning (Takara).

### 2.5 Growth curves of DHFR2 KO and DHFR KD cell lines

The growth of the DHFR2 knockout and DHFR knockdown cell lines was tested with and without supplementation (HT/NEAA). A wild‐type HepG2 cell line was used as a control and grown either in the presence or absence of supplementation. Each experiment was run in triplicate. Cell viability and number were monitored every three days for a total of ten days using the ADAM automated cell counter. Total and viable cells were counted, with only the latter considered in the growth analysis. The comparison of the cell growth of the cell populations was assessed via “compareGrowthCurves,” an algorithm included in the “statmod” package for statistical modeling in RStudio, created by Gordon Smyth.^20^, ^21^ This algorithm enables one to run pairwise comparisons between two or more groups of growth curves through a permutation test. A data frame is returned upon data submission containing the observed statistics (Stat), an estimated *p*‐value and an adjusted *p*‐value (for multiple testing). The data from the cell count were plotted into graphs to facilitate the comprehension of the growth trends of the tested cell lines and relative comparison. The data visualization package “ggplot2” was employed to produce all the graphs in RStudio.

### 2.6 PCR

Genomic DNA was purified using DNeasy Blood & Tissue kit (Qiagen) and quantified with NanoDrop One Microvolume UV–Vis Spectrophotometer (Thermo Fisher Scientific). Gene targets were amplified using Taq DNA Polymerase (5 U/μL, Sigma‐Aldrich). The primers used were as follows:

**Table:** 

DHFR2 Fb	5′‐TACCAAATGCGTGAAGACCA‐3′
DHFR2 Re	5′‐GGTTGTTCCATTGCACTCCG‐3′
MTHFD1 Fwd	5′‐CACTCCAGTGTTTGTCCATG‐3′
MTHFD1 Rev	5′‐GCATCTTGAGAGCCCTGAC‐3′
DHFR KO Fwd	5′‐GTCGCTGTGTCCCAGAAC‐3′
DHFR KO Rev	5′‐GCAGAAATCAGCAACTGGG‐3′
Invert cassette Fwd	5′‐CGCAGCAACAGATGGAAGG‐3′
Invert cassette Rev	5′‐AACTTGTGGCCGTTTACG‐3′
5#‐rev DonorVector	5′‐TGCTATGTAACGCGGAACTCCATATATGGG‐3′
3#‐fwd DonorVector	5′‐CAAACACGTACGCGTACGATGCTCTAGAATG‐3′
5Fwd DonorVect Mo	5′‐CCGCGTTACATAGCATCGTACGCGTACGTGTTTGGGACCTGCCCT
	GGCCACCGCTCCCCGGATCCATGGTGAGCAAGGG‐3′
3rev DonorVect Mo	5′‐ACGCGTACGTGTTTGGCCCCGGCCCGGCAGATACCTTCAGGCAC
	CGGGCTTGCG‐3′

### 2.7 RT‐PCR

Total cellular RNA was extracted using the PureLink RNA Mini Kit (Invitrogen) with on‐column DNase treatment. Possible DNA contamination was assessed via the MTHFD1 R653Q PCR assay before being reverse‐transcribed using the SuperScript III First‐Strand Synthesis SuperMix (Invitrogen). The primers used were as follows:

**Table:** 

FPDHFRL1Var1&2	5′‐CTTCCGGTAGCTGGTAAAGG‐3′
RPDHFRL1Var1&2	5′‐CCCATGTTTTGGGACACAG‐3′
DHFR Mains Fwd	5′‐CGCGAGCACGCCGCGACCCTGCGT‐3′
DHFR Mains Rev	5′‐CGCCCCCCTCGTCCCCATT‐3′

### 2.8 RT‐qPCR

RNA isolation and first‐strand synthesis were performed as described above. Complementary DNA (cDNA) was amplified by Fast Start Essential DNA Probes Master (Roche), in addition to primers and target‐specific probes from the Universal Probe Library Set, Human (Roche) in a Lightcycler 96 (Roche). mRNA abundance was quantified in relation to GAPDH, GUS, RPS13, or TBP as controls. The relative fold gene expression of samples was calculated using the 2–∆∆Ct method. Primers and probes used were as follows:

**Table:** 

GUS Fwd	5′‐GGTACGAACGGGAGGTGAT‐3′
GUS Rev	5′‐CACGATGGCATAGGAATGG‐3′
GUS Probe	73
GAPDH Fwd	5′‐CTCTGCTCCTCCTGTTCGAC‐3′
GAPDH Rev	5′‐ACGACCAAATCCGTTGACTC‐3′
GAPDH Probe	60
TBP Fwd	5′‐TTGGGTTTTCCAGCTAAGTTCT‐3′
TBP Rev	5′‐CCAGGAAATAACTCTGGCTCA‐3′
TBP Probe	24
RPS13 Fwd	5′‐GGTTGAAGTTGACATCTGACGA‐3′
RPS13 Rev.	5′‐TGTGCAACACCATGTGAATCT‐3′
RPS13 Probe	68
DHFR2_All Fwd	5′‐AATTTCGCGGCATTCTTG‐3′
DHFR2_All Rev.	5′‐GGTTAACACCTCCGAACTTGC‐3′
DHFR2_All Probe	72
DHFR Mains Fwd	5′‐CGCGAGCACGCCGCGACCCTGCGT‐3′
DHFR Mains Rev.	5′‐CGCCCCCCTCGTCCCCATT‐3′
DHFR Mains Probe	87
SHMT1 Fwd	5′‐TGGTGTAGAAATGGCCTCCT‐3′
SHMT1 Rev.	5′‐TCTCACACCAGGATGGGACT‐3′
SHMT1 Probe	58
SHMT2 Fwd	5′‐GATCCTGAGATGTGGGAGTTG‐3′
SHMT2 Rev.	5′‐CAGCTCGGCTGCAGAAGT‐3′
SHMT2 Probe	1
TYMS Fwd	5′‐CCCAGTTTATGGCTTCCAGT‐3′
TYMS Rev.	5′‐GCAGTTGGTCAACTCCCTGT‐3′
TYMS Probe	43
ALDH1L1 Fwd	5′‐ACCGCAACCTGACCTTGA‐3′
ALDH1L1 Rev.	5′‐TTCCAGGACAGCATCATCAG‐3′
ALDH1L1 Probe	12
SARDH Fwd	5′‐CACCGAGAGTGACCTGACTG‐3′
SARDH Rev.	5′‐GCCCATGGCCAGGTAGTA‐3′
SARDH Probe	83
tGFP Fwd	5′‐ACGATCTGGATGGCAGCTTC‐3′
tGFP Rev.	5′‐TCCACCACGGAGCTGTAGTA‐3′
tGFP Probe	41

### 2.9 Western blot

Cells were harvested at 80% confluency with Trypsin–EDTA 0.25% v/v (Gibco), washed with DPBS (Gibco), and resuspended in M‐PER Mammalian Protein Extraction Reagent supplemented with Halt Protease Inhibitor Cocktail EDTA‐Free (Thermo Fisher) as per manufacturer's protocol. The protein concentration was estimated via Bradford assay (Sigma Aldrich), and 25 μg of protein per sample was run on a Novex 4%–20% Tris‐Glycine gel (Invitrogen). The proteins were transferred onto a PVDF membrane and blocked in 5% (w/v) non‐fat milk diluted in 0.05% (v/v) TBST buffer. The membrane was incubated with primary antibody (anti‐β Actin (Cell Signalling Technology 8H10010), 1:1000 dilution; anti‐tGFP (Origene TA150071), 1:2000 dilution; anti‐DHFR (Abcam AB124814), 1:10 000 dilution) for 2 h and with a compatible secondary antibody. Immunoblotted proteins were detected using SuperSignal West Pico PLUS Chemiluminescent Substrate and imaged using Syngene's GeneGnome Bio Imaging System.

### 2.10 DHFR enzymatic assay

DHFR reductase activity was calculated based on NADPH consumption using a colorimetric assay (Abcam Dihydrofolate Reductase Assay Kit). The decrease in absorbance at 340 nm was measured in the Tecan I‐Control Infinite 200 spectrophotometer. The following equation was used to calculate DHFR activity as recommended by the manufacturer:

$$\begin{array}{c} \text{DHFR}\;\mathrm{mU}/\mathrm{mg}\\ ={\left(n\;\mathrm{mol}\;\text{sample}-n\;\mathrm{mol}\;\text{background}/\Delta t\right)}^{*}\;\mathrm{mg}\;\text{protein}\;\mathrm{per}\;\text{well}, \end{array}$$

where 1 U is the amount of DHFR that oxidizes 1 μmol NADPH per minute at pH 7.5 at room temperature.

### 2.11 Sanger sequencing

All PCR products were sequenced by either Source BioScience (Tramore, Ireland) or Eurofins Genomics (Ebersberg, Germany).

### 2.12 Folate metabolite profiling

Quantification of OCM intermediates was performed via ultraperformance liquid chromatography‐tandem mass spectrometry (UPLC–MS/MS), according to Ref. [22]. Cell pellets (circa 2 × 10^7^ cells/sample) were resuspended in a buffer containing 20 mM ammonia acetate, 0.1% ascorbic acid, 0.1% citric acid, and 100 mM DTT at pH 7. Cell suspensions were sonicated, and total proteins were extracted by precipitation with 2 volumes of acetonitrile. The proteins were lyophilized and stored at −80°C prior to mass spectrometry. Samples were rehydrated with 30 μL ultrapure water, centrifuged (at 12 000× *g*, 4°C, for 5 min), and transferred into glass vials for UPLC–MS/MS. Folate metabolites were resolved by reversed‐phase chromatography. Folate metabolites were eluted at a flow rate of 200 nL/min and run through a XEVO‐TQS mass spectrometer (Waters Corporation, UK) operating in negative‐ion mode (Capillary 2.5 kV, Source temperature 150°C, Desolvation temperature 600°C, Cone gas flow rate 150 L/h and Desolvation gas flow rate 1200 L/h). Folate intermediates were measured by Multiple Reaction Monitoring (MRM), using optimized cone voltage and collision energy for precursor and product ion. MassLynx software (Waters) was used to extract the peak areas of the individual folate metabolites.

### 2.13 RNA microarray

Total cellular RNA was extracted using PureLink RNA Mini Kit, including a DNase treatment (Invitrogen). Only the samples presenting A260/280 and A260/230 above 2 and concentrations above 1 μg/μl were considered eligible for microarray analysis. The mRNA/lncRNA expression profiling was performed by Arraystar Inc. (MD, USA) (Human LncRNA Array v5.0, Arraystar). RNA QC, cDNA synthesis, labeling, hybridization, scanning, and data analysis were performed by Arraystar according to their workflow. Impacted biological networks were investigated using the Qiagen IPA software.

### 2.14 Complementation and overexpression studies

Transient expression of DHFR2 was performed in HepG2 parental cells (overexpression) and DHFR2 knockout line (complementation) by transfecting the cells with pCMV6‐AC‐DHFR2 (RG232027, Origene), alongside pCMV6‐AC‐GFP (PS100010, Origene) as a control. Cells were seeded at 1.5 × 10^5^ cells/well in a 24‐well plate the day before transfection. Lipofectamine 3000 Transfection Reagent (Invitrogen) was used (as per manufacturer's protocol) along with 500 ng plasmid DNA per well. The lipid‐DNA complexes were assembled in Opti‐MEM and added to the cells, which were incubated (37°C, 5% CO_2_) for 48 h before being analyzed. The expression of the plasmid was assessed via RT‐qPCR and Western Blot. Expression profiles of the OCM genes were outlined using RT‐qPCR and mass spectrometry analysis.

### 2.15 LC–MS/MS analysis

LC–MS/MS analysis and profiling of digested protein samples was carried out using a Dionex Ultimate 3000 Reversed‐phase Capillary high‐pressure Liquid Chromatography (RSCLC) nanosystem coupled to a hybrid linear Ion‐trap/Orbitrap Fusion Tribrid mass spectrometer (Thermo Scientific, Dublin, Ireland). PepMap100 (C18, 300 μm × 5 mm) and Acclaim PepMap 100 (5 μm × 50 cm, 3 μm bead diameter) columns were used as the trapping and analytical columns, respectively. One microgram from each sample was separated for 120 min at 300 nL/min for data‐dependent analysis and 60 min at 300 nL/min for targeted MS analysis.

### 2.16 Data‐dependent acquisition (DDA) and targeted‐dependent acquisition (TDA) LC–MS/MS analysis

The DHFR KD cell line was examined for the presence of the DHFR2 peptide EAMNHLGHLK using the DDA and a TDA LC–MS/MS approach described in Bookey et al.^14^ The data‐dependent acquisition was carried out using a full scan range of 380–1500 m/z at 120 000 resolution at 200 m/z. MS2 scan conditions were set as follows: a targeted AGC value of 4 × 10^5^ and a maximum fill time of 50 ms. The number of selected precursor ions for fragmentation was determined using a top‐speed acquisition algorithm, and ions were isolated in the quadrupole using an isolation width of 1.6 Da. Dynamic exclusion was applied to the analyzed peptides after 60 s, and peptides with a charge state between 2+ and 6+ were analyzed. Fragmentation of precursor ions was carried out using higher energy collision‐induced dissociation (HCD) at 28% and the resulting MS/MS ions were detected via a linear ion trap. The AGC was set at 2 × 10^4^ with a maximum injection time set at 35 ms.

The TDA was carried out using a full scan range of 380–600 m/z at 120 000 resolution at 200 m/z. MS2 scan conditions were set as follows: a targeted AGC value of 4 × 10^5^ and a maximum fill time of 50 ms. The number of selected precursor ions for fragmentation was determined using a top‐speed acquisition algorithm and ions were isolated in the quadrupole using an isolation width of 0.7 Da. Fragmentation of precursor ions was carried out using higher energy collision‐induced dissociation (HCD) at 28% and the resulting MS/MS ions were detected via the orbitrap analyzer at 30 000 resolution at 200 m/z. The AGC was set at 5 × 10^4^ with a maximum injection time set at 300 ms. The DHFR2‐specific peptide (EAMNHLGHLK) had a retention time of 14 min and an m/z of 383.8668 for the double charge species and 575.2868 for the triply charged species.

### 2.17 Protein identification and differential protein expression profiling

Total protein was extracted from quadruplicate biological replicates and prepared for LC–MS/MS analysis according to Parle‐McDermott et al.^23^ LC–MS/MS analysis was carried out in NICB, DCU, Dublin, Ireland, and essentially as previously described.^24^

The Orbitrap Fusion Tribrid LC–MS/MS files were analyzed using Proteome Discoverer software version 2.2 (Thermo Scientific, Dublin, Ireland) using the SEQUEST HT algorithm against the Uniprot human database (fasta database downloaded in January 2022). A total of 10 ppm precursor mass tolerance and 0.6 Da fragment mass tolerance were used in all data‐dependent analysis searches, and 10 ppm and 0.02 Da for targeted MS analysis in Proteome Discoverer. Oxidation of methionine was set as a dynamic modification, and carbamidomethylation of cysteine was set as a static modification. A maximum of two missed cleavages was allowed during the search. A false discovery rate was set to be less than 5% using Percolator. Trypsin was set as the digestion enzyme for all samples. Progenesis QI for Proteomics (NonLinear Dynamics, Waters, Newcastle, UK) was used for quantitative label‐free LC–MS/MS differential expression analysis, and only proteins that passed the following criteria were considered differentially expressed between experimental groups: ANOVA *p*‐value of ≤.05; proteins with a minimum of 2 unique peptides contributing to the identification and a ≥1.5‐fold change in relative abundance. Network and protein function analysis was performed using the DAVID (https://david.ncifcrf.gov/) and STRING (https://string‐db.org/) databases.

### 2.18 Statistical analysis

Statistically significant differences in the relative expression ratios were assessed using a one‐way ANOVA test. The asterisks designate the statistical significance expressed by the *p*‐value as follows: *p ≤ .05, **p ≤ .01, ***p ≤ .001, ****p ≤ .0001, *****p ≤ .00001. Error bars shown in all graphs are standard deviations (*n* = 3) (SD).

## 3 RESULTS

### 3.1 RNA and proteomic profiling of DHFR2 KO reveals an impact on DNA replication/repair and one carbon metabolism

**FIGURE 1:**
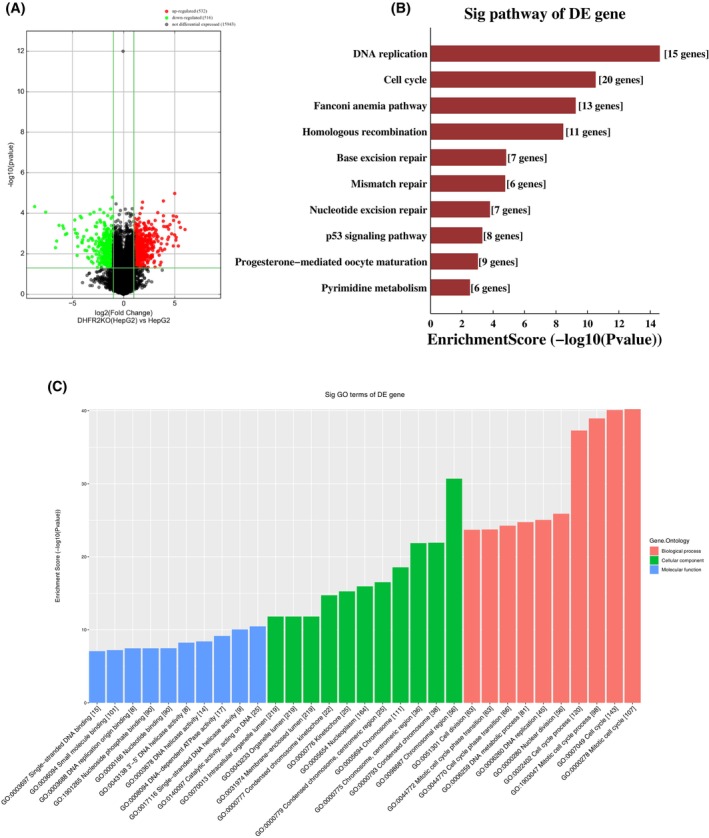
DHFR2 knockout differentially expressed genes. (A) Differentially expressed mRNAs with statistical significance were identified through Volcano filtering between DHFR2 KO and HepG2. The threshold is fold change *≥*2 and *p*‐value ≤.05. (B) Pathway analysis of the differentially expressed (DE) mRNAs in DHFR2 KO. Downregulated pathways with significant enrichment of DE genes. (C) GO analysis of the down‐regulated RNAs in DHFR2 KO (Bar Chart). The bar plot shows the top ten Enrichment Score values of the significant enrichment terms per each category: Biological Process in red, Cellular Component in green and Molecular Function in blue.

To understand the functional role of DHFR2 we sought to create and characterize a *DHFR2* gene knock‐out HepG2 model (DHFR2 KO). First, we analyzed the transcriptomic and proteomic profiles of the DHFR2 KO line. Global transcriptomics was investigated using microarray analysis from ArrayStar. The DHFR2 KO profile showed 1048 mRNAs differentially expressed (fold change ≥2.0 and *p* < .05), divided into 516 downregulated and 532 upregulated genes compared to the HepG2 wild‐type line (Figure 1A). Biological significance was assigned to the enriched genes using Gene Ontology and Pathway analyses. Of relevance are the downregulated gene sets, which showed increased enrichment scores (Figure 1B). Both GO and Pathway analyses illustrate that cell cycle and cell division suffer the most from DHFR2 loss, as do DNA replication, repair, and overall DNA metabolism. In line with these findings, nucleus and chromosomes were the cell components primarily affected by the DHFR2 KO (Figure 1C). We also assessed the cell proliferation rate of DHFR2 KO and compared it to its parental HepG2 line and found no significant difference (Figure S6).

We next assessed the DHFR2 KO proteome profile using label‐free LC–MS/MS quantitative proteomic analysis. The differential expression analysis identified 493 proteins, of which 272 were downregulated and 221 upregulated (Dataset Supporting Information S2 DE DHFR2KOvsWT—excel file). Impacted biological networks included (i) small molecule biochemistry, lipid metabolism, cell morphology; (ii) hereditary disorder, neurological disease, organismal injury and abnormalities; (iii) cellular assembly and organization, developmental disorder, and hereditary disorder. Furthermore, the STRING database was used to assess the differentially expressed protein sets more exhaustively, identifying that the most significantly affected pathways were de novo Thymidylate synthesis and the response to the DHFR inhibitor, Methotrexate. Other impacted pathways were glucuronate catabolic process to xylulose 5‐phosphate, de novo pyrimidine nucleobase biosynthetic pathway, fatty acid beta‐oxidation using acyl‐CoA oxidase, cardiac muscle cell adhesion, mitochondrial ribosome assembly, alditol catabolic process, and tetrahydrofolate metabolic process, that is, folate OCM. These findings suggest that the DHFR2 gene is functional and has relevance for a number of cellular pathways, primarily involving cell metabolism, DNA stability, and OCM.

### 3.2 Endogenous DHFR2 does not compensate for loss of DHFR expression

Previous LC–MS/MS proteomic analysis did not detect endogenous DHFR2 protein in any of the adult cell lines and tissues examined, including the parental wildtype HepG2 cell line.^14^ Therefore, we explored the hypothesis of DHFR2 providing reductase activity in the context of loss or impairment of DHFR activity. We generated a monoclonal cell line model in HepG2 cells that had reduced DHFR expression (referred to as DHFR KD). We assessed the cell proliferation rate of DHFR KD compared to its parental HepG2 line and found no significant difference (Figure S5). We also assessed whether there was upregulation of DHFR2 protein when DHFR levels were diminished. LC–MS offered a superior approach for distinguishing the two potential proteins and was performed on the DHFR KD HepG2 cells but neither DHFR nor DHFR2 specific peptides were detected in this experiment. (We detected DHFR peptides in the HepG2 parental, DHFR2 KO cells [and in previous proteomic experiments^14^]). Western blot analysis as the primary analytical method was problematic due to the difficulty of distinguishing DHFR and DHFR2 with antibodies. Despite this, we did run a Western blot with an anti‐DHFR antibody which detected a DHFR‐specific band in the DHFR KD (Figure S1), but it was at an abundance that was not detectable by LC–MS. However, given that DHFR was not fully knocked out in the DHFR KD line, we could not conclude that this band is DHFR2 and it is more likely to be residual DHFR. Overall, DHFR2‐specific peptides were not detected, meaning the lack of DHFR derived reductase activity did not induce an increase in the expression of the homologous DHFR2 protein (Dataset Supporting Information S1 DDA LC–MS analysis of DHFR KD‐excel file). A targeted LC–MS/MS method was also employed to specifically detect DHFR2 only. Finding a suitable candidate was challenging due to the high similarity between DHFR and DHFR2 (92% identity). Peptide EAMNHLGHLK was selected as suitable, despite having only one amino acid difference (Leu instead of Pro) with the DHFR relative peptide. The m/z ratio of the two versions of this peptide was significantly different to make it easy for the instrument to differentiate between them. The expected peptide mass and retention time information for DHFR/DHFR2 were used in this targeted analysis, and again the DHFR2 protein was not detected. It is notable that the instrument was able to identify the same peptide derived from two control lysates that consisted of either an expressed recombinant DHFR2 or spiked with the synthetic peptide. These experiments show that LC–MS/MS was unable to detect DHFR2‐specific peptides in several cell and tissue types, and in the context of a DHFR KD, despite a relative abundance of DHFR2 RNA isoforms in these cells.^1^, ^14^ It is possible, therefore, that its function is to be sought in the RNA form, but we cannot rule out that DHFR2 is present at an abundance that was not detectable with the methods we employed.

### 3.3 DHFR2 RNA levels have a direct impact on the enzymes involved in de novo Thymidylate synthesis, with a reduction in dihydrofolate reductase activity

**FIGURE 2:**
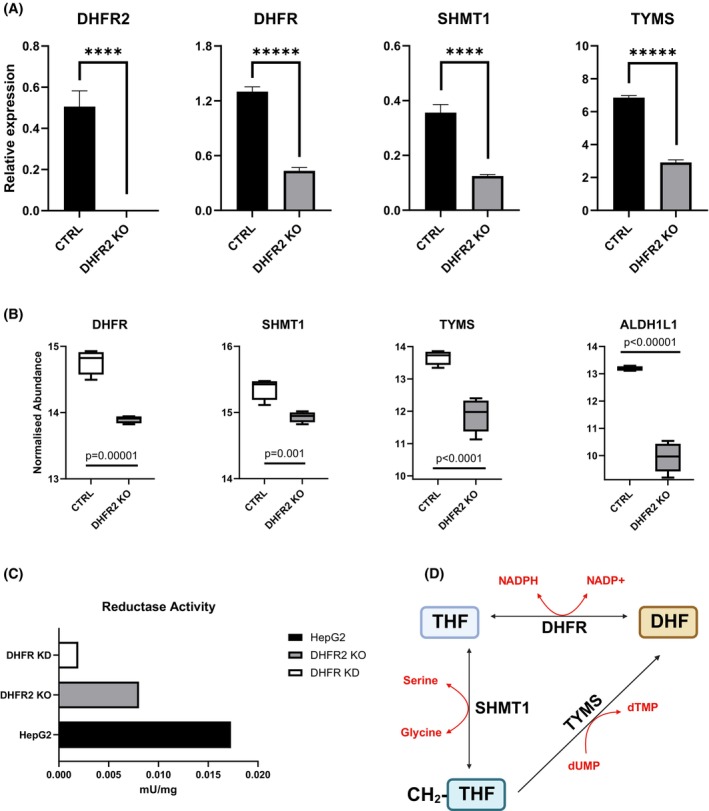
DHFR2 knockout impacts the enzymes of the de novo Thymidylate Synthesis pathway. (A) Endogenous levels of DHFR2, DHFR, TYMS, and SHMT1 mRNAs in DHFR2 KO compared to HepG2 parental/wildtype (CTRL) lines measured via RT‐qPCR. DHFR2 RNA is absent in the DHFR2 KO line, confirming the validity of the cell model. DHFR, TYMS and SHMT1 showed a drastic drop in expression compared to CTRL. Differences in relative expression ratios were compared using one‐way ANOVA.  Error bars show SD (*n* = 3). (B) Normalized abundance of DHFR, SHMT1, TYMS, and ALDH1L1 proteins within four replicates for the CTRL (HepG2 wt) and the DHFR2 KO cell lines. The expression of DHFR, SHMT1, TYMS, and ALDH1L1 proteins were all found to be significantly reduced in the DHFR2 KO. Statistical significance was calculated via Tukey's HSD test. Values are plotted as natural log (ln). (C) DHFR enzymatic assay. The DHFR activity to reduce NADPH was measured in HepG2, DHFR KD, and DHFR2 KO lines. The graph shows a significant reduction in activity of both edited lines, with DHFR2 KO and DHFR KD retaining 46% and 11% reductase activity, respectively. (D) Schematic of the de novo Thymidylate Synthesis Pathway.

Our transcriptome and proteome profiling indicated that KO of the *DHFR2* gene has an impact on many of the cellular pathways that are directly supplied by OCM, and therefore, we considered whether the enzymes of this pathway were also affected. In a separate experiment, the gene expression of specific enzymes of OCM was investigated using RT‐qPCR. We observed significant down‐regulation of *DHFR, SHMT1*, and *TYMS* expression levels in the DHFR2 KO when compared to the HepG2 wild‐type line. DHFR and SHMT1 relative expression declined threefold, while TYMS showed a twofold decrease in expression (Figure 2A). These three enzymes are all involved in de novo Thymidylate synthesis (Figure 2D).^3^, ^25^, ^26^ The transcriptome Array analysis correlated with the RT‐qPCR data; *SHMT1* and *TYMS* were significantly downregulated in the DHFR2 KO line (2.6 and 2‐fold, respectively) compared to the wild type. *DHFR* expression was not identified as a significantly differentially expressed gene on the array analysis, but its expression was also down.

We then examined the proteome profile of the DHFR2 KO line and compared it to the HepG2 wild‐type line using quantitative label‐free LC–MS/MS analysis to identify differentially expressed proteins belonging to the folate OCM pathway (Supporting Information S2 DE DHFR2KOvsWT—excel file). Interrogation of the proteome was consistent with the mRNA expression data outlined above (Supporting Information S2 DE DHFR2KOvsWT—excel file). The abundance of DHFR (2.40‐fold), SHMT1 (1.54‐fold), and TYMS (5.25‐fold) proteins was found to be significantly reduced in DHFR2 KO cells compared with the wild‐type parent line (Figure 2B). We also assessed dihydrofolate reductase enzyme activity in these cell lines and found that it was lowered (46%) compared to the wild‐type line (Figure 2C) based on a single experiment. We acknowledge that this enzyme activity experiment (Figure 2C) was limited due to constraints in the amount of protein available and does require further independent validation. However, the source of reductase enzyme activity in the DHFR2 KO line can only be derived from the parental homolog, DHFR, indicating that the expression of the *DHFR2* gene influences DHFR activity. The considerable downregulation of these three folate‐dependent enzymes suggests that loss of DHFR2 directly impacts de novo thymidylate synthesis through the regulation of its three main enzymes, DHFR, SHMT1, and TYMS (Figure 2D). Of note, a dramatic downregulation of aldehyde dehydrogenase 1 family member L1 (ALDH1L1) also occurred, that is, a reduction in the abundance of approximately 22‐fold (Figure 2B). This enzyme is a cytoplasmic 10‐formyltetrahydrofolate dehydrogenase and has a direct role in OCM by converting 10‐formyltetrahydrofolate to tetrahydrofolate.^27^, ^28^

### 3.4 DHFR2 knockout results in an altered OCM profile

**FIGURE 3:**
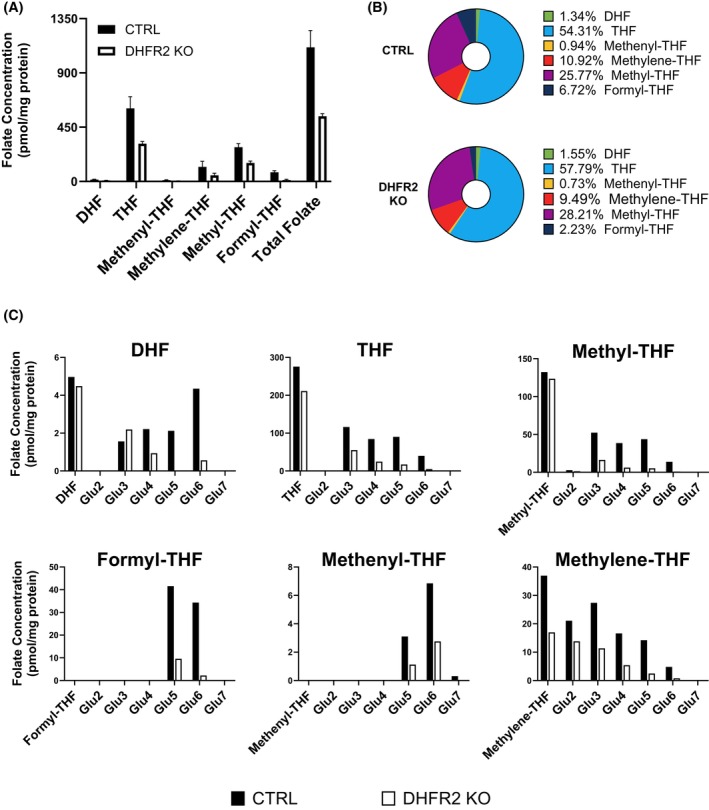
Folate metabolites profile in DHFR2 KO and HepG2 parental/wildtype (CTRL) lines. (A) The main forms of folate were measured in duplicate, with concentrations normalized to protein content and expressed as pmol/mg protein. All folate forms show halved concentration in DHFR2 KO compared to CTRL. (B) Relative proportions (expressed as % of total folate) of folate metabolites in DHFR2 KO and CTRL lines. The percentages for each folate intermediate were calculated considering the sum of all glutamated forms (n1–7). The proportions of the folate forms appear to be maintained with minor variations. (C) Concentration of folate intermediates and their relative polyglutamated forms in DHFR2 KO and CTRL lines. Glu2‐7 indicate the number of glutamate molecules attached. The concentrations were normalized to protein content and expressed as pmol/mg protein.

We next investigated the status of OCM intermediates. The major folate intermediates, including mono‐ and polyglutamated forms (2 to 7 glutamates attached), were quantified by LC–MS/MS (Figure 3A). This approach is sensitive to genetic disruption of individual steps in OCM and to inhibitors, including methotrexate.^29^ The percentage of each form of folate was calculated in relation to total folate, and folate concentration determined as pmol/mg protein. The abundance of the overall folate pool was diminished in DHFR2 KO compared with wild‐type HepG2, with the content approximately halved. However, in general, the proportions of folate species were maintained nonetheless (Figure 3B). The exception was 10‐formyl‐THF, which declined by roughly 20% in the DHFR2 KO line. A similar phenomenon is exhibited when considering polyglutamated forms of folate individually, with a significant decline in concentration of most forms in the DHFR2 KO line (Figure 3C). When the percentages of each intermediate are calculated in relation to total folate, it is evident that the proportions are comparable in HepG2 DHFR2 KO and HepG2 wild‐type folate pools, with slightly higher percentages of THF and 5′‐methyl‐THF, and a lower proportion of 10‐formyl‐THF in the DHFR2 knockout compared to the wild type (Figure 3B). The reduction in all forms of folate in the DHFR2 KO could be explained by the lowered dihydrofolate reductase activity that we observed in DHFR2 KO cells that appears to be driven by the downregulation of DHFR. This is supported by our measurements of folic acid concentrations in the cells using ion count data. We observed a lower abundance but not statistically significant difference of unmetabolized folic acid concentrations between DHFR2 KO compared to the HepG2 parental (wildtype line) (Figure S7). The lower abundance of folates observed in the DHFR2 KO is likely to be due to lower DHFR activity and subsequently, less folic acid entering OCM. These results suggest that the loss of DHFR2 may result in a general impairment of overall OCM.

### 3.5 DHFR2 elicits a direct regulation on DHFR

**FIGURE 4:**
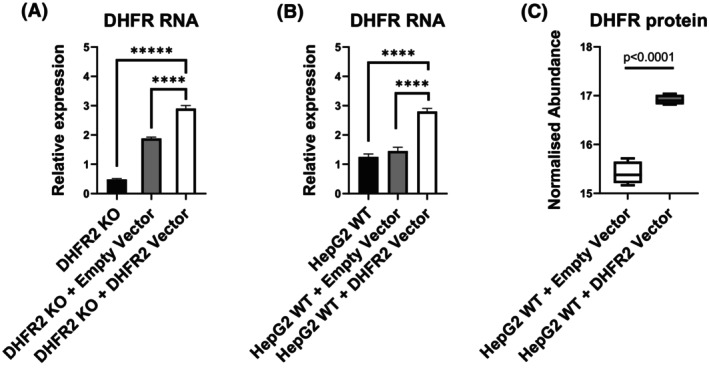
DHFR2 elicits a direct response on *DHFR* RNA expression. (A) Relative expression of *DHFR* RNA in DHFR2 KO line and upon transfection with pCMV6‐AC‐GFP (EV, empty vector) and pCMV6‐AC‐DHFR2 (DHFR2 ORF, vector containing DHFR2 ORF) measured via RT‐qPCR. *DHFR* expression significantly increases in relation to the restored DHFR2 expression when compared to the DHFR2 KO line and DHFR2 KO + EV. (B) Relative expression of *DHFR* RNA in the DHFR2 HepG2 wild‐type overexpression model measured via RT‐qPCR. *DHFR* RNA levels rise in response to an increase in DHFR2 expression (+DHFR2 ORF), suggesting a proportional regulation of *DHFR* in relation to the *DHFR2* expression levels. (C) MS‐based differential expression analysis in DHFR2 HepG2 wild‐type overexpression model. Normalized abundance of DHFR protein within four replicates for the HepG2 transfected with pCMV6‐AC‐GFP (+EV, empty vector) and pCMV6‐AC‐DHFR2 (+DHFR2 ORF). Similarly to the *DHFR* RNA expression, DHFR2 overexpression is followed by an increase in DHFR protein. Differences in relative expression ratios (RT‐qPCR) were compared using one‐way ANOVA. Error bars show SD (*n* = 3). Statistical significance of the MS‐based differential expression was plotted as Tukey's HSD test, with values expressed as natural log (ln).

A genetic complementation experiment was conducted to assess if a DHFR2‐expressing transgene could rescue the DHFR2 KO phenotype and restore the normal expression of the genes involved in de novo thymidylate synthesis. Our DHFR2 KO line was transfected with either a recombinant DHFR2‐tGFP clone under the control of a CMV promoter or the empty vector. Recombinant expression was assessed via fluorescence microscopy (for detection of GFP) (Figure S2), RT‐PCR and RT‐qPCR (Figure S3), Western blot (Figure S4) and Mass Spectrometry. DHFR2‐tGFP expression was relatively low compared to the empty vector control. We observed peak recombinant protein expression (despite the application of puromycin selection) on Day 2 post‐transfection, which drastically dropped by Day 9. Therefore, RNA and protein extracts from Day 2 samples were used to assess the complementation ability of recombinant DHFR2. Of the three genes found to be downregulated in DHFR2 KO cells, *DHFR*, *TYMS*, and *SHMT1*, only *DHFR* exhibited a significant increase in RNA levels (Figure 4A). However, this RNA increase was accompanied by neither an equally significant rise in the DHFR protein nor SHMT1 or TYMS. These results suggest that the main target of DHFR2 may be DHFR, which it primarily modulates at the RNA level.

We also overexpressed recombinant DHFR2 in the HepG2 wild‐type line. DHFR relative expression was significantly raised compared to both HepG2 wild‐type and HepG2 empty vector (Figure 4B). Unlike the complementation experiment, the mass spectrometry analysis revealed an equivalent increase in the DHFR enzyme (Figure 4C). However, SHMT1 and TYMS did not show any significant differential expression. These findings suggest that the direct regulatory target of DHFR2 is only DHFR, with SHMT1 and TYMS possibly modifying their expression in response to DHFR level.

### 3.6 DHFR knockdown results in upregulation of DHFR2 RNA suggesting a feedback mechanism

**FIGURE 5:**
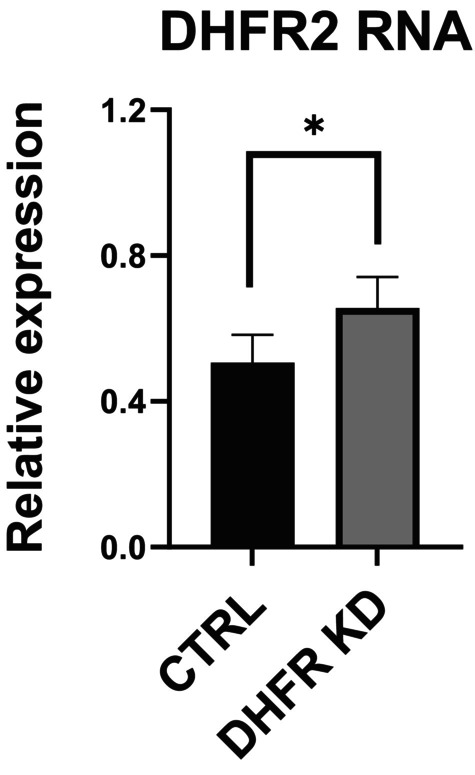
DHFR KD results in the upregulation of DHFR2 RNA. Endogenous levels of DHFR2 mRNAs in DHFR KD compared to HepG2 parental/wildtype (CTRL) lines measured via RT‐qPCR. DHFR2 showed a moderate expression increase in DHFR KD compared to CTRL. Differences in relative expression ratios were compared using one‐way ANOVA. Error bars show SD (*n* = 3).

Our results indicate that DHFR2 RNA may directly regulate DHFR. To test if a reciprocal regulation of DHFR2 by DHFR also occurs, we investigated the expression levels of DHFR2 in a DHFR KD line. Our DHFR KD line appears to produce an unstable DHFR protein, which accounts for the retained 10% of reductase activity compared to the wild‐type line (Figure 2C). The examination of *DHFR2* relative expression in the DHFR KD model revealed a moderate but significant increase in RNA levels (Figure 5). These data provide additional evidence for a regulatory interaction between DHFR2 and DHFR, with interdependence on each other's expression levels.

## 4 DISCUSSION

**FIGURE 6:**
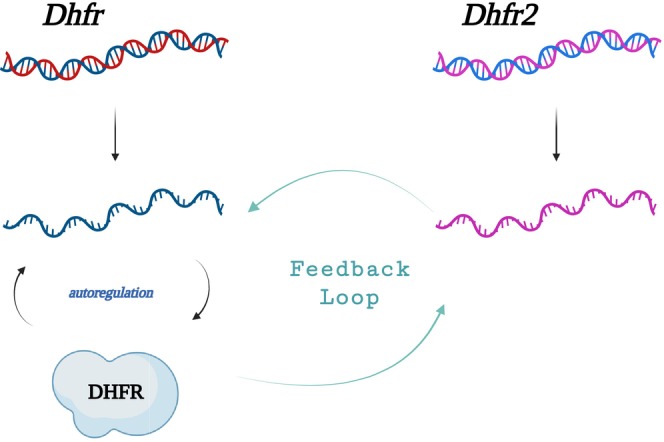
Proposed model of DHFR/DHFR2 feedback loop regulation. DHFR2 RNA regulates DHFR RNA (and protein, consecutively). Low levels of DHFR enzyme trigger an increase in DHFR2 which, in turn, leads to a DHFR RNA rise. DHFR enzyme levels are intrinsically regulated by the protein's ability to bind its own RNA in an auto‐regulatory mechanism. DHFR2 RNA might be involved in the autoregulatory mechanism or act as a “sponge” for the DHFR protein that impacts its autoregulation.

We have confirmed that while *DHFR2* RNA isoforms are relatively abundant across a range of cells and tissue types^2^ (*H. sapiens* DHFR2 ensg00000178700 Expression Atlas^30^), these mRNAs are not translated into a DHFR2 protein that is detectable using a variety of proteomic methodologies as described in this paper (Dataset Supporting Information S1 and S2) and that of Bookey et al.^14^ Despite this, we show that the *DHFR2* gene does have relevance for folate OCM as modulation of its expression has an impact on total folate levels and their associated metabolite profile. Knockout of *DHFR2* results in a global decrease in abundance of folates when compared to the parental HepG2 wildtype cell line (Figure 3). Folate metabolic enzymes are saturated in the cell leading to competition for substrates. It has been suggested that nucleic acid synthesis is prioritized over other reactions such as methylation, and this appears to be also evident in DHFR2 knockout cells, where a drop in 10‐formyltetrahydrofolate abundance and, to a lesser extent, 5,10‐methylenetetrahydrofolate was observed. 10‐formyltetrahydrofolate provides two 1Cs for purine synthesis,^29^, ^31^, ^32^, ^33^ and 5,10‐methyleneTHF is the 1C donor for de novo thymidylate synthesis via SHMT.^25^, ^34^ We also observed a dramatic downregulation of the 10‐formyltetrahydrofolate dehydrogenase enzyme (ALDH1L1) in our DHFR2 KO cells. This further supports the hypothesis that nucleic acid synthesis is the priority of OCM in our cell line model, as its downregulation will ensure that 10‐formyltetrahydrofolate remains available for DNA synthesis. ALDH1L1 silencing has been shown to confer a metabolic advantage for tumor progression as it supports cell proliferation.^35^ The functional impact of *DHFR2* and its effect on OCM and nucleotide synthesis is further emphasized by the observed impact of knockdown on a number of cellular pathways, including DNA replication and repair, and cell‐cycle regulation at both the RNA and protein levels. These pathways are directly affected by OCM, which supplies the 1C units required for purine and pyrimidine synthesis.^36^, ^37^ The specific impact of *DHFR2* RNA expression modulation on DHFR and its associated partner enzymes for de novo thymidylate synthesis, that is, SHMT1 and TYMS (Figure 2D), is likely to be one of the main drivers that are influencing these pathway changes. Our *DHFR2* RT‐qPCR analysis showed upregulation of the *DHFR2* transcript in the DHFR KD (Figure 5), indicating that a possible regulatory mechanism is in place to compensate for the drop in the observed dihydrofolate reductase activity in this cell line model (Figure 2C). We speculate that the decline in DHFR activity triggers the up‐regulation of a DHFR2 RNA, which, being a direct modulator of DHFR, induces an increase in DHFR expression. This mechanism would close the regulatory cycle, creating a feedback loop where DHFR is regulated by DHFR2, which in turn is modulated by DHFR (Figure 6). The exact mechanism of how DHFR is regulated by DHFR2 is not clear, but it is worth noting that the DHFR protein is capable of translation autoregulation through binding to its own mRNA. It is also capable of binding to DHFR2 mRNA,^2^ and these interactions may feature in how DHFR2 mediates its influence on DHFR expression from mRNA to protein. One possible mechanism is that DHFR2 RNA acts as an alternative binding site for the DHFR protein (a “sponge”) and dilutes the amount of protein available to inhibit translation of DHFR via autoregulation. However, the impact on both mRNA and protein levels that we observed might not correlate with such a “sponge” model whereby stalled translation may not necessarily impact mRNA abundance. However, mRNA degradation may increase due to stalled translation providing a hypothetical explanation for our data. Of note, our experiments were conducted on a hepatocyte cancer cell line (HepG2 and their gene edited derivatives), and these results may not be applicable to noncancerous hepatocyte cells or indeed to other cell types or tissues.

The DHFR2 KO cell line had a lower dihydrofolate reductase activity compared to the wild type (Figure 2C), supporting our proposal that *DHFR2* RNA directly regulates DHFR. Moreover, our RT‐qPCR data correlates with our proteomics data in the DHFR2 knockout line compared to the wild type (Figure 2; Supporting Information S2), which shows a significant downregulation in the RNA and protein levels of the three enzymes required for de novo thymidylate synthesis, that is, DHFR, SHMT1, and TYMS (Figure 2D). SHMT1 converts serine to 5,10 methylene‐THF by transferring a 1C group from the amino acid to a molecule of THF. The methylene‐THF is the substrate of TYMS, which passes the activated 1C to dUMP, thus creating dTMP. This reaction releases DHF, which is again converted into THF by DHFR, closing the cycle.^26^ Upon SUMOylation, the three enzymes translocate to the nucleus and assemble into a dTMP synthesis complex (dTMP‐SC). The dTMP‐SC has been found anchored to the nuclear lamina and in DNA replication sites, providing DNA precursors on‐site.^38^, ^39^, ^40^, ^41^, ^42^ This might be especially important in preventing uracil misincorporation^39^, ^43^, ^44^, ^45^ and subsequent DNA damage.^46^ The complex has been seen migrating in the nucleus during DNA replication and repair,^47^ but it was also found in the cytoplasm.^48^ dTMP synthesis was also observed in mitochondria, although using the mitochondrial isozyme SHMT2.^1^ Therefore, we can associate the DHFR2 RNA regulatory function to the nuclear/cytoplasmic de novo dTMP synthesis pathway since all three enzymes of the complex undergo significant down‐regulation due to *DHFR2* gene removal. When we complemented the DHFR2 KO line by overexpressing recombinant DHFR2, we observed a restoration of DHFR expression levels after 48 h post‐transfection; similarly, the overexpression of recombinant DHFR2 in the HepG2 wild‐type line resulted in a significant increase in DHFR expression at both RNA and protein level (Figure 4). SHMT1 and TYMS did not show a recovery of their expression in the same experiments in the 48‐h time‐frame, and this suggests that DHFR2 RNA is regulating DHFR directly. Given that endogenous DHFR2 protein was not detected in wild‐type parental HepG2 cells, the effects that we are observing are most likely to be due to one or more of the many RNA isoforms of the *DHFR2* gene (Ensembl DHFR2 ENSG00000178700).^49^ Further evidence supporting a regulatory interaction between DHFR and DHFR2 arises from our DHFR KD model, which shows a moderate upregulation of *DHFR2* RNA compared to the wild type (Figure 5). Our results are consistent with that of Anderson et al.,^1^ who initially proposed DHFR2 as the mitochondrial reductase. Their DHFR2 (referred to as DHFRL1) siRNA knockdown had an impact on mitochondrial de novo thymidylate synthesis, and the current data indicate that this effect was being driven by the impact of DHFR2 RNA on DHFR regulation and not due to downregulation of a DHFR2 protein. DHFR appears to mediate dihydrofolate reductase activity in both the mitochondrial and cytosolic compartments. However, it is not yet known whether diminished DHFR activity resulting from impaired expression of DHFR or DHFR2 alters the relative contribution of folate OCM in each compartment to outputs such as thymidylate biosynthesis. Future work could address this question by isotope labelling, for example using [2,3,3‐2H] serine.^50^, ^51^

We propose a regulatory feedback model of how *DHFR2* RNA interacts and regulates cellular dihydrofolate reductase enzyme activity through its interactions with DHFR mRNA and protein (Figure 6). Given that one of the main functions of *DHFR2* is as a regulatory RNA molecule, we suggest that it is a long noncoding RNA (lncRNA).^52^ The definition of a long noncoding RNA (lncRNA) is an RNA of longer than 200 nucleotides that does not code for a protein. This relatively new category of RNA is diverse, complex, and our understanding of their functionality is still evolving. Ultimately, whether a given gene or one of its RNA isoforms should be classified as a lncRNA relates to the binary classification of RNAs as coding or noncoding to date. It is likely that genes produce many RNA products that have diverse functions that deviate from the central dogma and how these are classified remains to be clarified. Although some lncRNAs are defined as lacking an apparent ORF, we propose that DHFR2 warrants the title of lncRNA as we have found no evidence for production of an endogenous protein (Supporting Information S1)^14^ and the impact of its expression level on DHFR. In conclusion, we propose that the *DHFR2* gene encodes a lncRNA that directly regulates cellular dihydrofolate reductase activity through its impact on DHFR mRNA and protein abundance. *DHFR2* RNA represents a novel clinical target that has potential to enhance current and future anti‐folate therapies.

## AUTHOR CONTRIBUTIONS

Anne Parle‐McDermott and Nicholas D. E. Greene conceived the study. Paola Drago, Niamh Bookey, Nicholas D. E. Greene, and Anne Parle‐McDermott designed the study. Paola Drago designed the gene edited and overexpression cell model construction with Anne Parle‐McDermott and Niamh Bookey and executed the experimental work including all RNA expression analyses and DHFR enzyme activity assays. Niamh Bookey designed all proteomics analyses with Anne Parle‐McDermott, Paola Drago, Michael Henry, and Paula Meleady and executed the experimental work with support for LC–MS/MS from Paula Meleady, Michael Henry, Nicholas D. E. Greene, and Kit‐Yi Leung designed and performed the folate metabolite analyses. All authors contributed to data interpretation. Paola Drago and Anne Parle‐McDermott wrote the first draft of the manuscript.

## DISCLOSURES

The authors do not have any competing interests.

## Supporting information


Data S1.

Dataset S1.

Dataset S2.

## Data Availability

All the data in this manuscript are contained within it or as Supporting Information. The RNA profiling microarray data are also available at ArrayExpress Collection in Biostudies (https://www.ebi.ac.uk/arrayexpress/) (Accession number: E‐MTAB‐12958). The primers used are available at PrimerBank (http://pga.mgh.harvard.edu/primerbank/) (DOI: https://doi.org/10.25504/FAIRsharing.8bwhme). The proteomics profiling data are available via ProteomeXchange with the identifier PXD044513.
