# Supplementary material for: DHFR2 RNA directly regulates dihydrofolate reductase and its expression level impacts folate one carbon metabolism
Source: FASEB J. 2025 Feb 17;39(4):e70391. doi: 10.1096/fj.202401039RR (PMC11831416; doi:10.1096/fj.202401039RR)
Supplement: Supplementary file 1 — Data S1. [file FSB2-39-e70391-s003.docx]

**Supplementary Information for**

**DHFR2 RNA directly regulates Dihydrofolate Reductase and its expression level impacts folate One Carbon Metabolism**

Paola Drago, Niamh Bookey, Kit-Yi Leung, Michael Henry, Paula Meleady, Nicholas D.E. Greene, Anne Parle-McDermott.

Prof. Anne Parle-McDermott

Email: [anne.parle](mailto:xxxxx@xxxx.xxx)-mcdermott@dcu.ie

**This PDF file includes:**

Supplementary text

Figures S1 to S7

Legends for Datasets S1 to S2

SI References

**Other supplementary materials for this manuscript include the following:**

Datasets S1 (SI 1. DDA DHFRKD.xlsx) to S2. (SI 2. DE DHFR2KOvsWT.xlsx)

**Supplementary Information Text**

The RT-qPCR, RT-PCR and Western blot Methods were performed as described in the main text.

**Methods**

**Growth Curves of DHFR2 KO and DHFR KD cell lines**

The growth of the DHFR2 knockout and DHFR knockdown cell lines were tested with and without supplementation (HT/NEAA). A wild-type HepG2 cell line was used as a control and grown either in the presence or absence of supplementation. Each experiment was run in triplicate. Cell viability and number were monitored every three days for a total of ten days using the ADAM automated cell counter. Total and viable cells were counted, with only the latter considered in the growth analysis. The comparison of the cell growth of the cell populations was assessed via ‘compareGrowthCurves’, an algorithm included in the ‘statmod’ package for statistical modelling in RStudio, created by Gordon Smyth (Baldwin et al., 2007; Elso et al., 2004). This algorithm enables one to run pairwise comparisons between two or more groups of growth curves through a permutation test. A data frame is returned upon data submission containing the observed statistics (Stat), an estimated p-value and an adjusted p-value (for multiple testing). The data from the cell count was plotted into graphs to facilitate the comprehension of the growth trends of the tested cell lines and relative comparison. The data visualisation package ‘ggplot2’ was employed to produce all the graphs in RStudio.

**
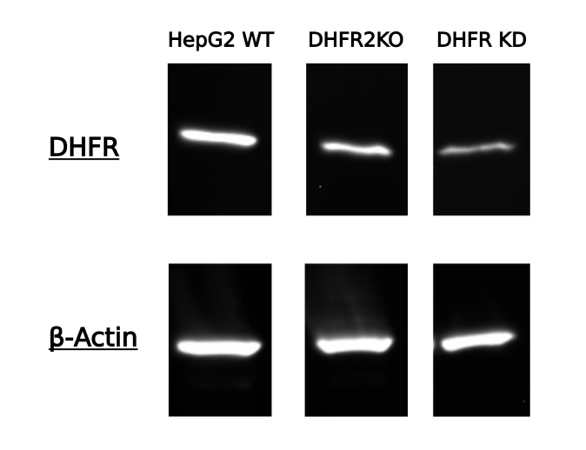
**

**Fig. S1. Confirmation of DHFR KD by Western blot.** Western blot with Anti-DHFR antibody (ABCam ab1248814) and Anti-β-Actin antibody (CST 8H10D10) in HepG2 wildtype (WT) cells, DHFR2 Knockout (KO) and DHFR Knockdown (KD). A band of approximately 22kDa was detected with the DHFR antibody while a band of approximately 45kDa was detected with the β-Actin antibody. Western blots were performed as described in the main text.


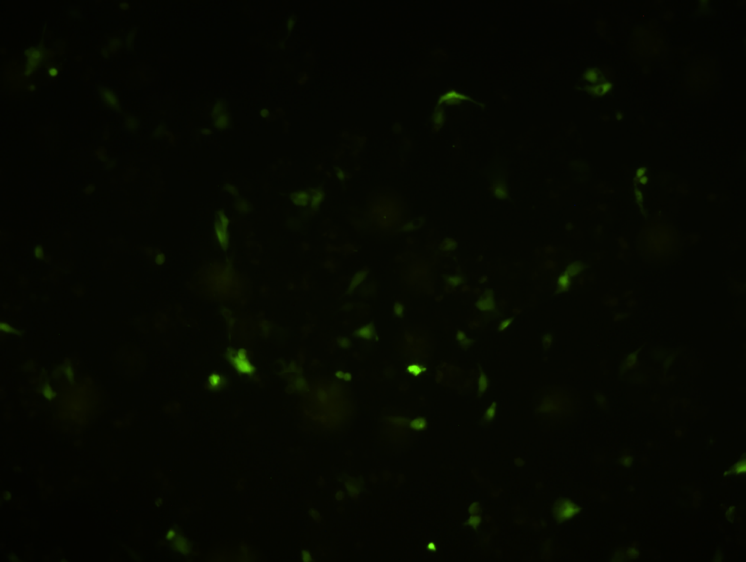

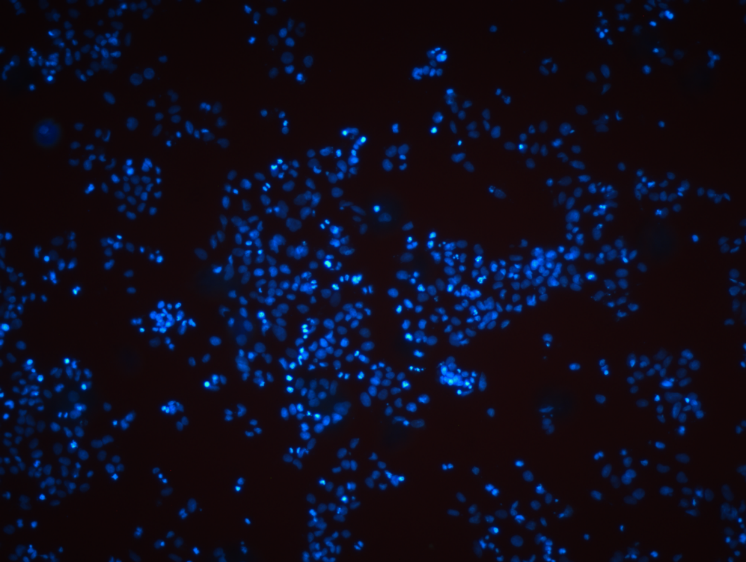
DHFR2KO + pCMV6-AC-GFP (control) _ Day 2 post-transfection


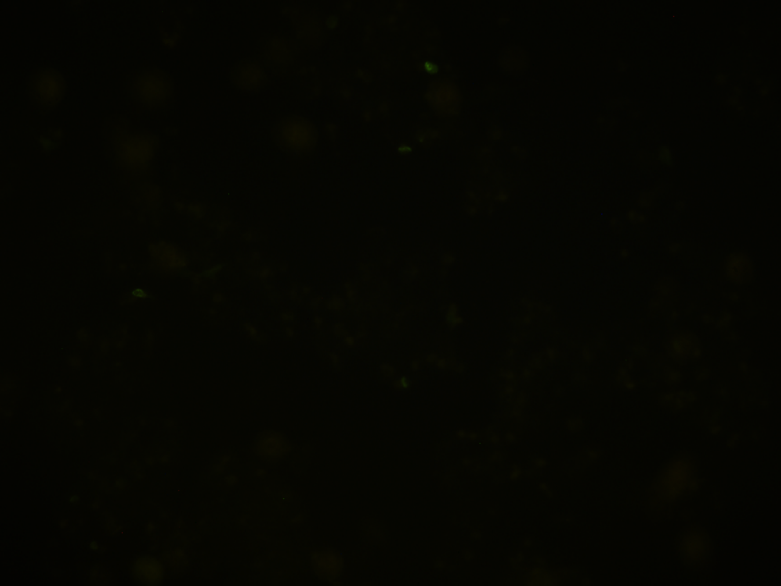

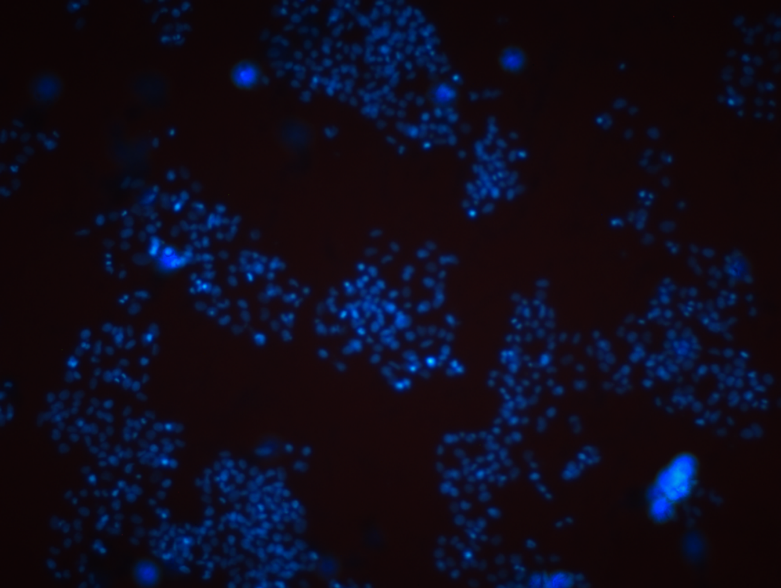
DHFR2KO + pCMV6-AC-DHFR2-GFP _ Day 2 post-transfection


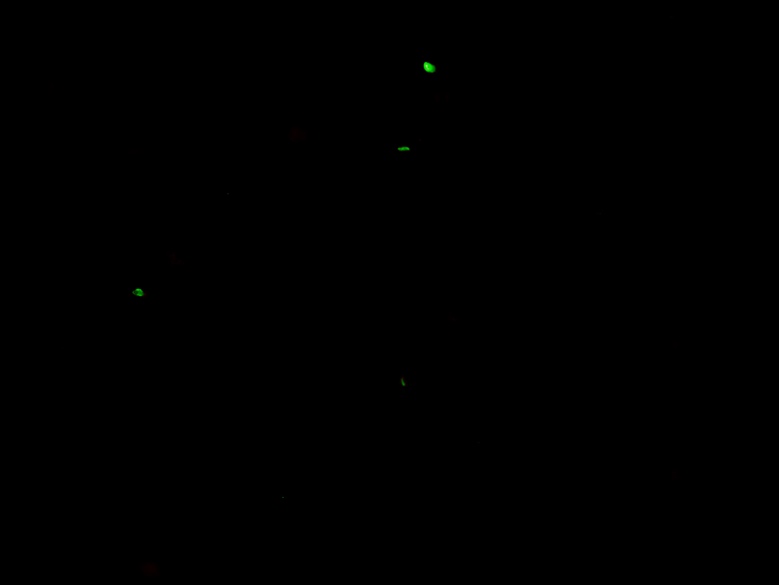


With increased exposure

**Fig. S2. Recombinant DHFR2-GFP expression in DHFR2 KO line by fluorescent microscopy.** Cells transfected with pCMV6-AC-DHFR2-GFP or pCMV6-AC-GFP (control) were fixed and counter-stained with DAPI. Fluorescence was observed using a Leica DFC 500, and pictures were taken via the Leica Application Suite software. Top two panels = empty vector (DHFR2KO + pCMV6-AC-GFP (control). Middle two panels = DHFR2-GFP (DHFR2KO + pCMV6-AC-DHFR2-GFP). Bottom panel = DHFR2-GFP with a longer exposure. Recombinant DHFR2-GFP protein was observed but at a low level.


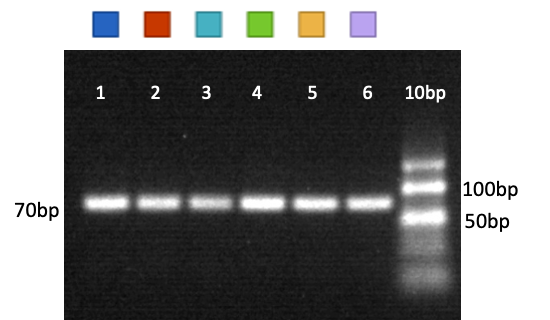


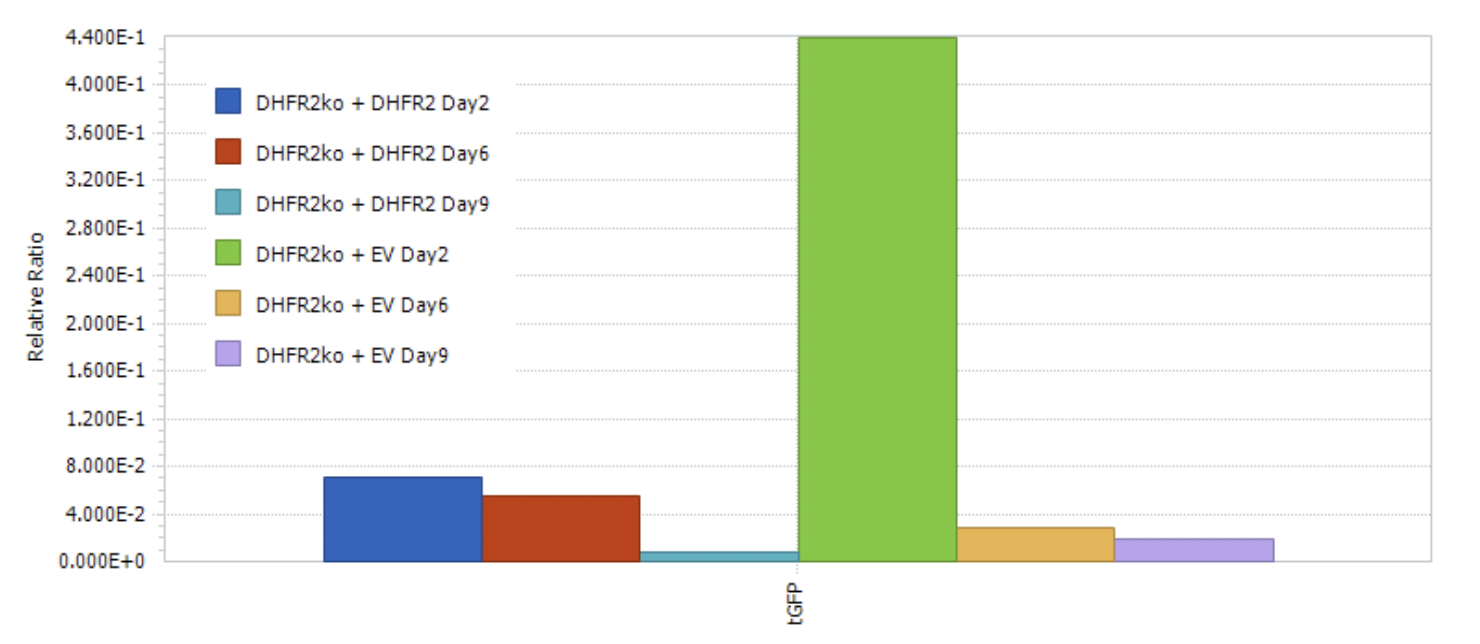


**Fig. S3. GFP mRNA expression levels in DHFR2 KO cells transfected with recombinant DHFR2-GFP and assessed by RT-PCR (top panel) and RT-qPCR (bottom panel).** Recombinant DHFR2-GFP mRNA expression in DHFR2 knockout line (DHFR2ko). RNA samples were isolated on 2- (dark blue), 6- (red), and 9 (light blue) days post-transfection. Controls are the DHFR2ko line transfected with empty vector and RNA isolated on 2- (green), 6- (orange) and 9-(purple) days post-transfection. DHFR2ko GFP RNA expression was confirmed by RT-PCR, and relative quantification was normalised to GAPDH.


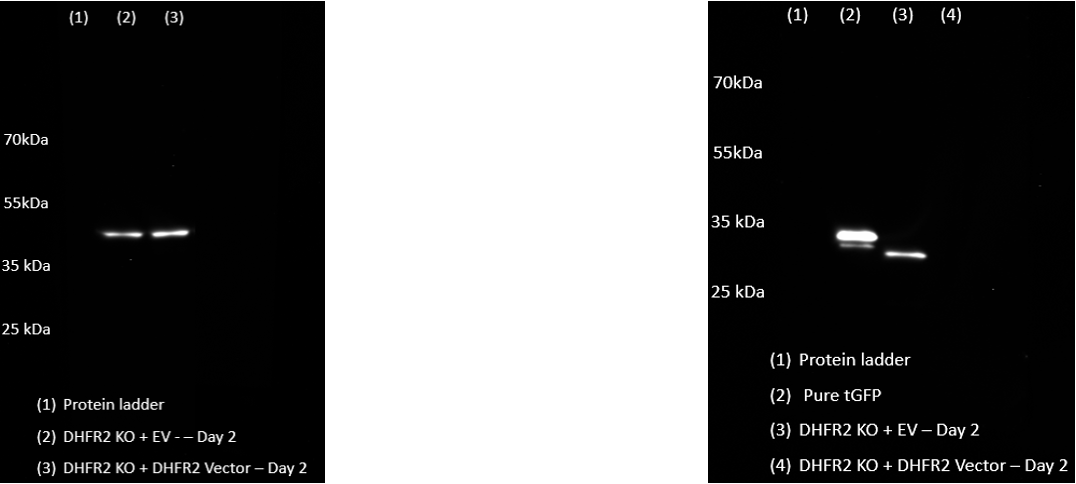


β-Actin

tGFP

**Fig. S4. Western blot analysis of recombinant DHFR2-GFP protein expression in DHFR2 KO line.** Western blot analysis with anti-tGFP (right panel) in DHFR2 knockout line transfected with either pCMV6-AC-DHFR2-GFP or empty vector pCMV6-AC-GFP. Purified recombinant tGFP was included as a positive control, and β-Actin as a loading control (left panel). A total of 25 μg of each sample was loaded in each lane. Origene tGFP antibody (TA150071), CST β-Actin antibody (8H10010). Recombinant DHFR2-GFP protein was not detected using an anti-tGFP antibody.


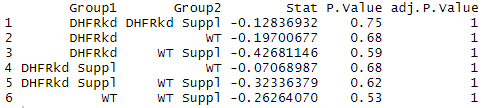

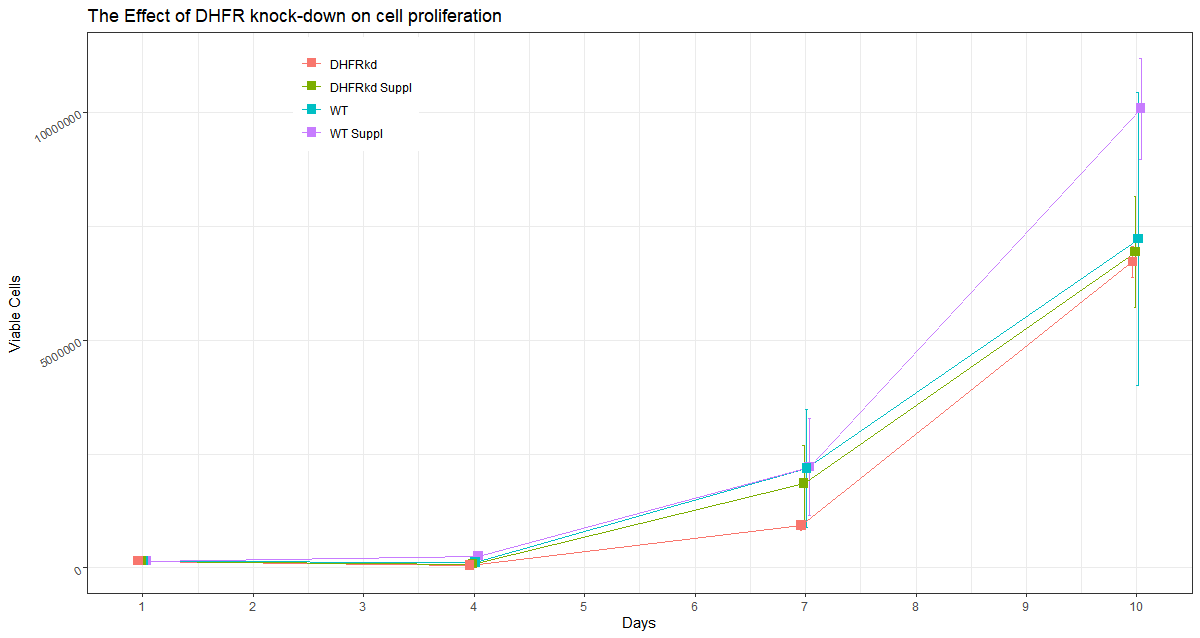


*5b.*

*5a.*

**Fig. S5 DHFR KD Growth Curves**

a. Permutation test for pairwise comparisons. Test run between groups of growth curves using the algorithm ‘compareGrowthCurves’ in RStudio. DHFRkd, HepG2 DHFR Knock-down line; WT, HepG2 wild-type line; Suppl, Hypoxanthine + Thymidine (HT) / Non-essential Amino acids (NEAA) supplementation. No significant differences in growth rate was observed.

b. Growth curves of HepG2 DHFR knock-down line compared to HepG2 wild-type. Cellular growth curves determined by PI (Propidium Iodide) staining method combined with advanced image analysis by the automated fluorescence cell counter ADAM-MC. All measures were performed in triplicate. DHFRkd, HepG2 DHFR Knock-down line; WT, HepG2 parental line; Suppl, Hypoxanthine + Thymidine (HT) / Non-essential Amino acids (NEAA) supplementation.

*6a.*

*6b.*


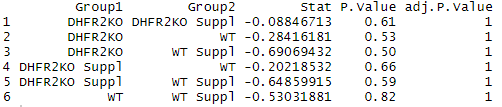

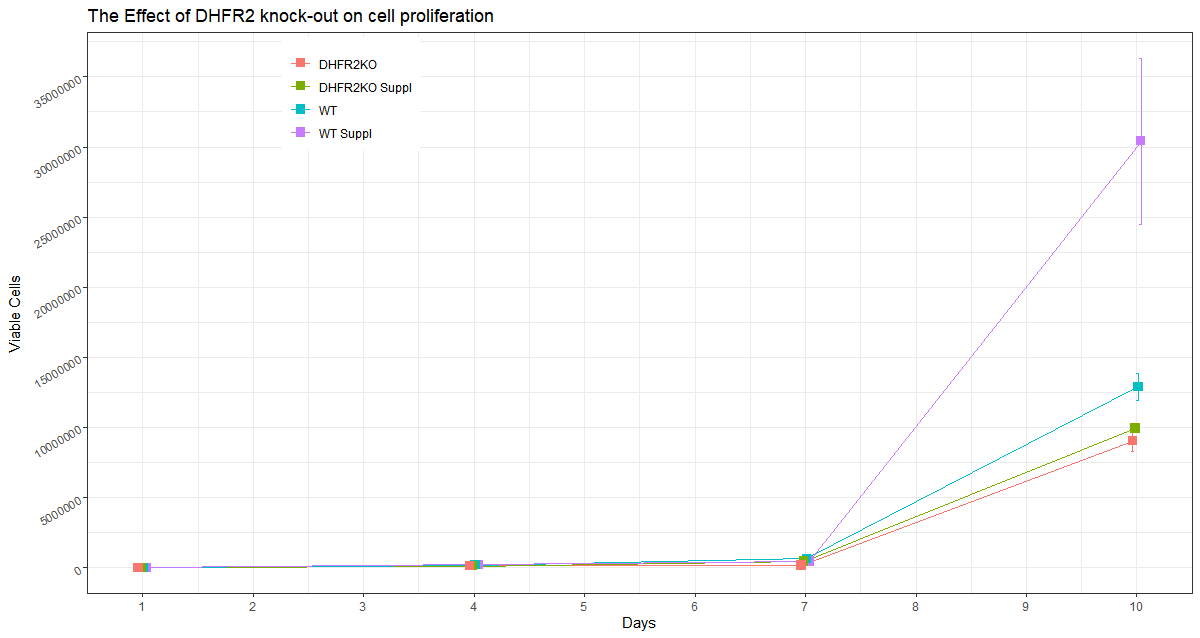


**Fig. S6. DHFR2 KO Growth Curves**

a. Permutation test for pairwise comparisons. Test run between groups of growth curves using the algorithm ‘compareGrowthCurves’ in RStudio. DHFR2KO, HepG2 DHFR2 Knockout line; WT, HepG2 wild-type line; Suppl, Hypoxanthine + Thymidine (HT) / Non-essential Amino acids (NEAA) supplementation. No significant differences in growth rate was observed.

b. Growth curves of HepG2 DHFR2 knockout line compared to HepG2 wild-type Cellular growth curves determined by PI (Propidium Iodide) staining method combined with advanced image analysis by the automated fluorescence cell counter ADAM-MC. All measures were performed in triplicate. DHFR2KO, HepG2 DHFR2 Knockout line; WT, HepG2 wild-type line; Suppl, Hypoxanthine + Thymidine (HT) / Non-essential Amino acids (NEAA) supplementation.

**­**

**Fig. S7. Comparison of folic acid abundance between HepG2 wild type (WT) and DHFR2 knockout (KO) cells using UPLC-MS/MS.** The amount of folic acid does not differ significantly in HepG2 WT and DHFR2 KO (T-test, p=0.304).

**Dataset S1 (separate file). Data Dependent Acquisition HepG2 DHFR KD LC-MS/MS proteomics data file.**

**Dataset S2 (separate file). Differential Proteomics expression profile comparison of HepG2 DHFR2 KO versus HepG2 parental/wildtype by LC-MS/MS data file.**

**SI References**

Baldwin, T., Sakthianandeswaren, A., Curtis, J. M., Kumar, B., Smyth, G. K., Foote, S. J., & Handman, E. (2007). Wound healing response is a major contributor to the severity of cutaneous leishmaniasis in the ear model of infection. Parasite Immunology, 29(10), 501–513.

Elso, C. M., Roberts, L. J., Smyth, G. K., Thomson, R. J., Baldwin, T. M., Foote, S. J., & Handman, E. (2004). Leishmaniasis host response loci (lmr1-3) modify disease severity through a Th1/Th2-independent pathway. Genes and Immunity, 5(2), 93–100. https://doi.org/10.1038/sj.gene.6364042
